# Beyond Lumping and Splitting: A Review of Computational Approaches for Stratifying Psychiatric Disorders

**DOI:** 10.1016/j.bpsc.2016.04.002

**Published:** 2016-09

**Authors:** Andre F. Marquand, Thomas Wolfers, Maarten Mennes, Jan Buitelaar, Christian F. Beckmann

**Affiliations:** a Donders Centre for Cognitive Neuroimaging, Donders Institute for Brain, Cognition and Behaviour, Radboud University, Nijmegen; b Department of Cognitive Neuroscience , Radboud University Medical Centre, Nijmegen; c Karakter Child and Adolescent Psychiatric University Centre, Nijmegen, The Netherlands; d Department of Neuroimaging (AFM), Centre for Neuroimaging Sciences, Institute of Psychiatry, King’s College London, London; e Oxford Centre for Functional Magnetic Resonance Imaging of the Brain (CFB), University of Oxford, London, United Kingdom

**Keywords:** European Roadmap for Mental Health Research, Heterogeneity, Latent cluster analysis, Psychiatry, RDoC, Research Domain Criteria, Subgroup, ROAMER

## Abstract

Heterogeneity is a key feature of all psychiatric disorders that manifests on many levels, including symptoms, disease course, and biological underpinnings. These form a substantial barrier to understanding disease mechanisms and developing effective, personalized treatments. In response, many studies have aimed to stratify psychiatric disorders, aiming to find more consistent subgroups on the basis of many types of data. Such approaches have received renewed interest after recent research initiatives, such as the National Institute of Mental Health Research Domain Criteria and the European Roadmap for Mental Health Research, both of which emphasize finding stratifications that are based on biological systems and that cut across current classifications. We first introduce the basic concepts for stratifying psychiatric disorders and then provide a methodologically oriented and critical review of the existing literature. This shows that the predominant clustering approach that aims to subdivide clinical populations into more coherent subgroups has made a useful contribution but is heavily dependent on the type of data used; it has produced many different ways to subgroup the disorders we review, but for most disorders it has not converged on a consistent set of subgroups. We highlight problems with current approaches that are not widely recognized and discuss the importance of validation to ensure that the derived subgroups index clinically relevant variation. Finally, we review emerging techniques—such as those that estimate normative models for mappings between biology and behavior—that provide new ways to parse the heterogeneity underlying psychiatric disorders and evaluate all methods to meeting the objectives of such as the National Institute of Mental Health Research Domain Criteria and Roadmap for Mental Health Research.

Psychiatric disorders are, without exception, highly heterogeneous in terms of symptoms, disease course, and biological underpinnings. Diagnoses are made on the basis of symptoms, while at the level of the underlying biology their causes are complex and multifaceted. This becomes acutely problematic in psychiatry because biological tests to assist diagnosis or predict outcome have not been developed (1). Diagnostic categories therefore often do not map cleanly onto either biology or outcome, which forms a major barrier to understanding disease mechanisms and developing more effective treatments.

A recognition of the imperfections of psychiatric nosology is not new; the debate between “lumpers” and “splitters” (2) over the number and validity of diagnostic classifications has continued unabated for more than a century following the classifications of dementia praecox and schizophrenia proposed by Kraepelin and Bleuler (3, 4). Reflecting this ongoing debate, classifications are revised with every new edition of diagnostic manuals (5, 6). Data-driven approaches to address heterogeneity in psychiatric disorders have also been applied for decades, in which the dominant approach has been to partition clinical groups into more homogeneous subgroups using data clustering methods—early examples can be seen in Paykel (7) and Farmer *et al.* (8). These approaches have recently received renewed interest for three reasons: 1) the advent of technologies for measuring many aspects of biology noninvasively and in vivo, particularly neuroimaging and genetics; 2) advances in statistical and machine learning data analytic approaches that make it possible to extract information from complex and high-dimensional data; and 3) increasing emphasis on using biological data to tailor treatments to the needs of individual patients (“precision medicine”) (9, 10). Most notably, recent funding initiatives, such as the National Institue of Mental Health Research Domain Criteria [RDoC (11)] and the European Roadmap for Mental Health Research [ROAMER (12)], have encouraged researchers to think beyond the classical case-control approach—where participants are either “patients” or “controls” based on fixed diagnostic criteria—and instead link cognitive dimensions with underlying biology while cutting across diagnostic classifications. The hope is that this will lead to biologically grounded understanding of disease entities and ultimately to more effective, personalized treatments.

These initiatives have stimulated an increasing number of studies that have used data-driven methods to stratify many disorders, including schizophrenia, major depression, attention-deficit/hyperactivity disorder (ADHD), and autism based on many types of data, including symptoms, neuropsychologic scores, and neuroimaging measures (13, 14, 15, 16, 17, 18, 19, 20, 21). We selectively review this burgeoning literature.^1^ We first present a didactic introduction to the most prevalent methodologic approaches for stratifying psychiatric disorders, highlighting the (often implicit) assumptions they entail. We then present an illustrative overview of studies that have used these methods to parse the heterogeneity underlying psychiatric disorders. We identify problems with current approaches and discuss the importance of validation to ensure reproducibility and ensure that clusters map onto clinically meaningful variation. We discuss emerging techniques, such as normative modeling (22), that provide means to parse heterogeneity in clinical cohorts without needing to make strong assumptions about clinical groups and evaluate the suitability of each method for meeting the objectives of recent research initiatives. Finally, we propose future developments that may help to parse heterogeneity more effectively.

## Methodologic Approaches For Stratifying Clinical Populations

The predominant approach has been to subdivide clinical cohorts using statistical or machine learning methods, largely of two main types: clustering (23) and finite mixture models (FMMs) (24, 25, 26). Both are unsupervised in that they do not have access to class labels (e.g., diagnostic labels) and must find subgroups automatically based on structure within the data and heuristics used by each algorithm. In contrast, supervised methods are provided with labels that indicate the class to which each subject belongs (e.g., “patient” or “control”). Supervised learning has been successful for predicting diagnosis or outcome from neuroimaging data in research settings (27, 28, 29) but is fundamentally limited by the quality of the clinical labels and the heterogeneity within disease cohorts (29) and cannot, by definition, inform on the validity of the labels. Therefore, unsupervised methods have been more widely used for discovering latent structure within clinical groups. We present a brief introduction to clustering and FMM methods below; additional details and a didactic introduction are provided in the Supplement.

### Clustering

The classical case-control approach can itself be phrased in terms of defining clusters and associated decision boundaries. For example, Fisher’s linear discriminant (23) uses the class-dependent mean response (e.g., in patients vs. controls) and thereby clusters the entire cohort along a decision boundary defined by the mean and class-specific covariances. More generally, given a set of data points (e.g., clinical or neuroimaging measures), clustering algorithms aim to partition the data into a specified number (K) of clusters such that the samples in each cluster are more similar to one another than to those in the other clusters. This entails defining a measure of similarity or distance between data points. One of the simplest and most widely used approaches is K-means clustering, which partitions the input space into K subregions based on the squared Euclidean distance (see Supplement). A wide variety of other algorithms have also been proposed in the machine learning literature (23, 30, 31). Two that are relevant for stratifying psychiatric disorders are 1) hierarchical clustering, which forms a hierarchy of cluster assignments by recursively splitting larger groups (“divisive clustering”) or combining individual samples (“agglomerative clustering” [e.g., Ward’s method (32)]), and 2) community detection, which is a graph-based method that aims to cluster nodes into “communities” (33).

### Finite Mixture Modeling

FMMs^2^ are a broad class of probabilistic approaches that aim to represent data using a finite number of parametric distributions (“components”). The simplest examples are Gaussian mixture models (GMMs),^3^ where all components have Gaussian distributions (24), but many other models are also members of this class (26), including latent class cluster analysis (LCCA) (25, 34), growth mixture modeling (35), latent class growth analysis^4^ (LCGA) (36), and factor mixture modeling (20) (see Supplement).

LCCA is a particularly widely used approach that accommodates many different data types (e.g., continuous, categorical, and ordinal). It is highly generic and can model, for example, dependence between variables (e.g., to model correlated clinical variables) or can use covariates to help predict class membership (25, 26, 34). Growth mixture modeling is a useful generalization and is derived by combining FMM with growth models (26, 35). This is appropriate for modeling longitudinal data derived from different growth trajectories. Given the neurodevelopmental basis for psychiatric disorders (37) and the importance of disease course in diagnosis (38), these approaches are increasingly being applied to stratify psychiatric disorders (39, 40).

One advantage of FMMs is that they provide a full statistical model for the data, and therefore classical statistical techniques can be used to assess fit (e.g., likelihood ratio tests). They are also flexible; for example, GMMs can approximate any continuous distribution to acceptable error (41). However, modeling complex distributions may require many mixture components having many parameters.

### Model Order Selection

Choosing the number of clusters or components is an important consideration and directly influences model flexibility. Many techniques have been proposed for comparing model orders, including classical information criteria (42, 43) and specialized methods (44, 45, 46, 47, 48). Different methods embody different heuristics (e.g., how parameters are penalized), which may not yield the same or even a unique optimal model order, indicating that the data can be equally well-explained using different model orders. Some methods automatically estimate model order (33, 49) but do not indicate whether other model orders are equally appropriate and often have additional parameters that influence the estimated model order. For example, graph-based methods (33) entail specifying a threshold above which nodes are considered connected (see Advantages and Disadvantages of Clustering for further discussion).

## Applications to Stratify Psychiatric Disorders

Clustering methods^5^ have been used extensively to stratify all psychiatric disorders, both individually and across diagnoses; Table 1, Table 2, Table 3, Table 4, Table 5 provide a representative (but not exhaustive) overview. Several articles offer more extensive quantitative reviews (19, 50, 51). Three salient observations can be made: first, during the many years that computational approaches have been used, relatively few algorithms have been used. There is, however, more variability among methods to select model order. Second, stratifications have been based on a range of measures, but predominantly symptoms or psychometric variables. This is notable considering that RDoC and ROAMER emphasize stratification on the basis of mappings between biological systems and cognitive domains, not just symptoms (10). To date, few studies have stratified psychiatric disorders on the basis of quantitative biological measures, and these studies have predominantly used neuroimaging-based measures (13, 16, 17, 52). This may be because of well-known problems with clustering complex, high-dimensional data (see Advantages and Disadvantages of Clustering).

## Clinical Implications

One of the most striking features evident from Table 1, Table 2, Table 3, Table 4, Table 5 is that the outcomes of clustering are heavily dependent on the input data; the overall picture derived from the literature is a profusion of different ways to subtype psychiatric disorders with relatively little convergence onto a coherent and consistent set of subtypes (19, 50). The disorder with the most consistent stratifications across studies is major depression, where many (53, 54, 55, 56), but not all (57, 58, 59) studies report evidence for “typical” (melancholic) and “atypical” subtypes, although these often do not align with the classical DSM subtypes (60). In contrast, stratifications of schizophrenia, ADHD, and autism have been much more variable across studies. In these cases, it is difficult to know how these different clustering solutions relate to each other or which are most relevant for clinical decision-making. From a clinical perspective, the discrepancies in these findings may reflect different subgroupings being reflected in different measures or a convergence of multiple causal mechanisms on the same phenotype. There are hundreds of genetic polymorphisms associated with most psychiatric disorders (61, 62), all having small effect sizes and converging on similar symptoms. This aggregation of small effects has been likened to a “watershed,” where genetic polymorphisms aggregate as they flow downstream, finding full expression in the syndromic expression of the disorder (63). An additional complication in comparing studies is that symptom profiles of many disorders vary over the course of the disorder, even within individual subjects (64). Therefore, quantitative comparisons between different studies and cohorts are needed, as is a greater focus on external validation (see below).

## Advantages and Disadvantages of Clustering

Table 1, Table 2, Table 3, Table 4, Table 5 show that clustering algorithms have been the method of choice for stratifying clinical groups and have made an important contribution to studying the heterogeneity underlying psychiatric disorders. Clustering methods are ideal if the disorder can be cleanly separated into subgroups (e.g., for separating typical from atypical depression). However, our review shows that psychiatric disorders cannot be reproducibly stratified using symptoms alone, probably because of extensive overlap between disorders. Indeed, finding an optimal solution is in general a computationally difficult problem (65).^6^ Therefore, all algorithms used in practice use heuristics to find approximate solutions that do not guarantee convergence to a global optimum. This is not overly problematic in itself, and standard approaches are to run multiple random restarts to find the best solution possible or to integrate different solutions to provide measures of cluster uncertainty. A more serious problem is that clustering algorithms always yield a result and partition the data into the specified number of clusters regardless of the underlying data distribution (Supplementary Figure S1). The number and validity of the clusters must be specified a priori or assessed post hoc. In this regard, it is important to recognize that different approaches to clustering embody different heuristics, possibly leading to different solutions. These heuristics are determined by many factors, including the choice of algorithm and distance function, the model order, the subspace in which clustering takes place, and the method used to search the space. Moreover, in general it is not possible to adjudicate unambiguously between methods because there is no clear measure of success for unsupervised learning methods (23).^7^ For example, different metrics for assessing model order often yield different answers and also may not identify a unique optimal model order. Therefore, heuristics and previous expectations play a strong role in the choice of algorithm and model order. Indeed, many studies use multiple approaches, aiming for consensus (Table 1, Table 2, Table 3, Table 4, Table 5), but the final choice of method is often a matter of taste.

High-dimensional data bring additional problems for clustering that are well-recognized in the machine learning literature (see Supplementary Methods) (31,66). Specialized algorithms are therefore recommended for high-dimensional data (31, 66), but to date these have not been applied to psychiatric disorders. Another problem for biological data (e.g., neuroimaging and genetics) is that the magnitude of nuisance variation is usually larger than clinically relevant variation, so the clustering solution can be driven by the nuisance variation rather than clinical heterogeneity. Therefore, it can be difficult to constrain clustering algorithms to find clinically relevant clusters, which necessitates careful data handling and preprocessing.

More specific problems with applying clustering algorithms to stratify psychiatric disorders include the following: 1) some participants may not clearly belong to any class; 2) some classes may be not well defined or may be unmanageably small (67); 3) subgroups may principally index severity (39, 55, 68); and 3) it is not clear whether healthy participants should be clustered separately or in combination with patients.

## Validation

The complexity of deriving clustering solutions makes validation crucial to ensure reproducibility and to ensure that the derived clusters index clinically meaningful variation. A common approach is to train supervised classifiers to separate classes using the same data that were used to derive the clusters or data that are highly correlated (e.g., different symptom measures). However, this approach is circular and simply measures how well classes can be separated within the training sample. A better approach is to assess cluster reproducibility, which requires additional cohorts or resampling of the data (e.g., cross-validation). However, to avoid bias, the entire procedure—including clustering—must be embedded within the resampling framework. To assess clinical validity, external data are necessary and should be defined a priori. For this, prediction of future outcome is considered the best test (69) if outcome can be clearly defined (e.g., the absence of relapse in schizophrenia). Biological measures can also provide useful validation because they can determine whether clusters map onto pathophysiology (11, 12), which is important because subgroups that reduce phenotypic heterogeneity may not reduce biological heterogeneity (70). Historically, the importance of validation has been somewhat overlooked (Table 1, Table 2, Table 3, Table 4, Table 5), but it is reassuring to note that studies are increasingly validating stratifications against external measures, especially in the case of major depression (60, 71, 72, 73); for example, Rhebergen *et al.* (39) derived a set of symptom trajectories to stratify depressed subjects that were subsequently validated against measures of affective processing derived from functional magnetic resonance imaging scans (73). Another notable example of external validation was provided by Karalunas *et al.* (14), who stratified children with ADHD on the basis of temperament ratings and validated these stratifications against cardiac measures, resting state functional magnetic resonance imaging scans, and clinical outcome.

## Alternatives to Clustering

**Figure 1:**
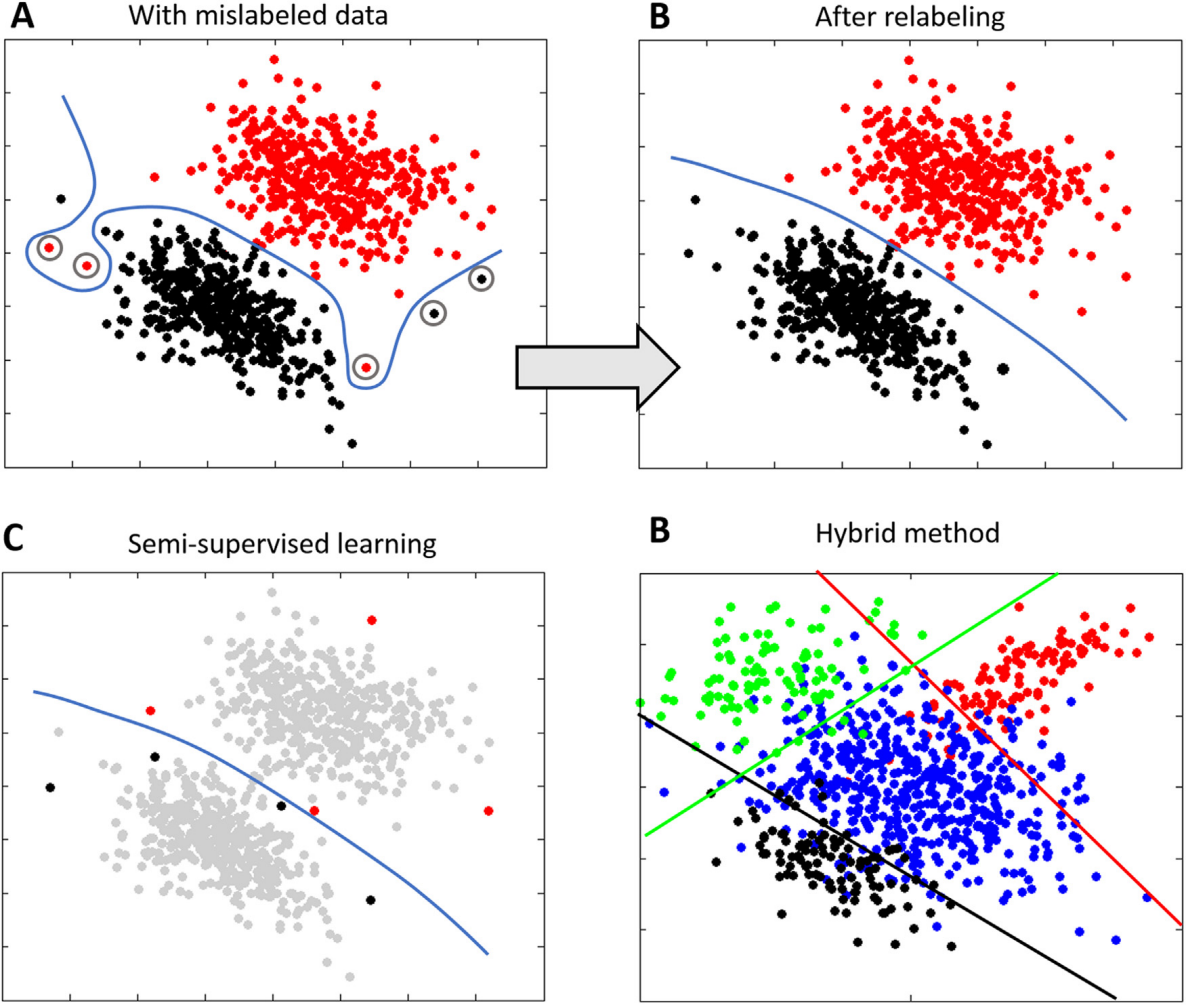
Schematic examples of alternative approaches to clustering and finite mixture models based on supervised learning. **(A)** This example shows the benefit of correcting mislabeled training samples. A supervised classifier trained to separate experimental classes (black and red points) may be forced to use a complex nonlinear decision boundary (blue line) to separate classes if data points are mislabeled (circled). **(B)** A simpler decision boundary results if the incorrect labels are corrected, for example using a wrapper method (74). **(C)** In a semisupervised learning context (75), only some data points have labels (black and red points). These can correspond to samples for which a certain diagnosis can be obtained. All other data points are unlabeled, but can still contribute to defining the decision boundary. Hybrid methods (76, 77, 78) combine supervised classification with unsupervised clustering and use multiple linear decision boundaries to separate the healthy class (blue points) from putative disease subgroups (colored points). See text for further details.

**Figure 2:**
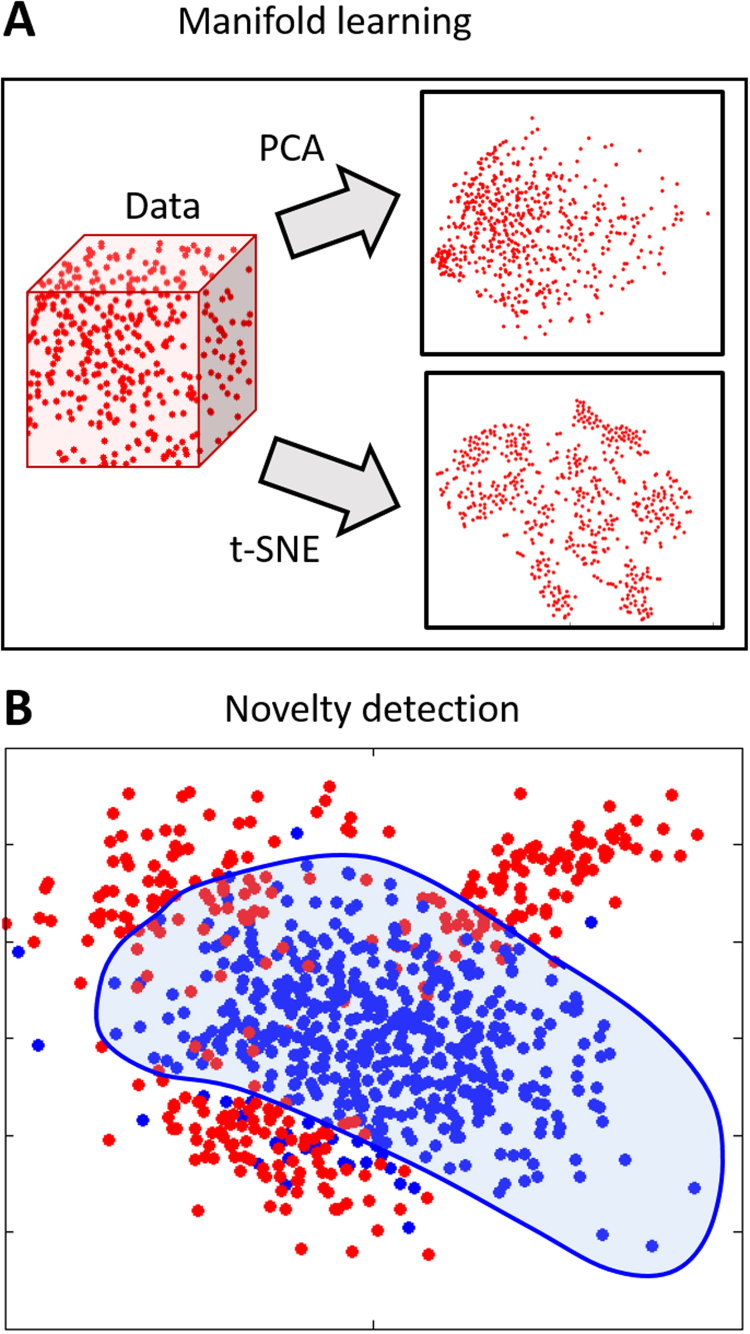
Schematic examples of alternative approaches to clustering and finite mixture models based on unsupervised learning. **(A)** Manifold learning techniques aim to find some low-dimensional manifold (right panels) that represent the data more efficiently than the original high-dimensional data (depicted by the cube on the right). Basic dimensionality reduction techniques, such as principal components analysis (PCA), find a single subspace for the data based on maximizing variance. This may not efficiently show structure in high-dimensional data. In contrast, approaches that preserve local distances, such as t-stochastic neighbor (t-SNE) embedding (80), may highlight intrinsic structure more effectively. **(B)** Novelty detection algorithms, such as the one-class support vector machine (83), aim to find a decision boundary that encloses a set of healthy subjects (blue points), allowing disease profiles to be detected as outliers (red points). Note that this approach does not provide an estimate of the probability density at each point.

**Figure 3:**
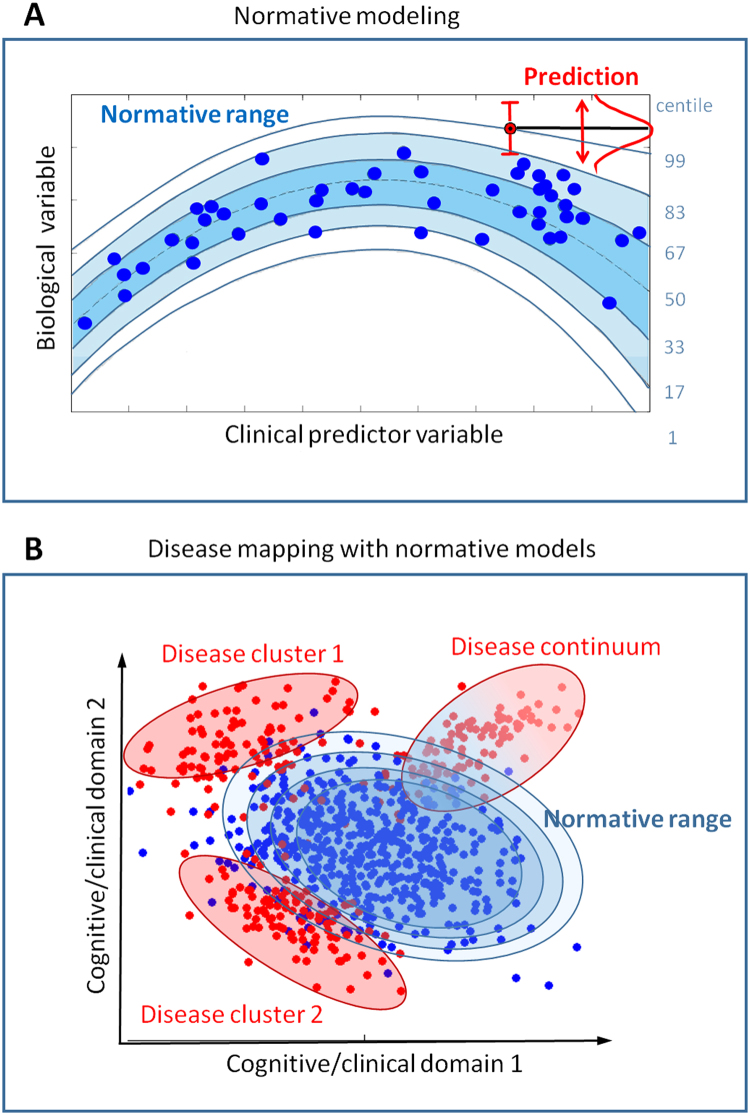
**(A)** Normative modeling approaches (22, 85, 86) aim to link a set of clinically relevant predictor variables with a set of quantitative biological response variables while quantifying the variation across this mapping. This is achieved by estimating a nonlinear regression model that provides probabilistic measures of predictive confidence (blue contour lines). These could be certainty estimates derived from a probabilistic model (22) or classical confidence intervals (86) and can be interpreted as centiles of variation within the cohort (blue numerals, right). Predictions for new data points (red) can then be derived that provide measures of predictive confidence to quantify the fit of the new data point to the normative model. [Adapted with permission from (22).] **(B)** By performing this mapping across different domains of functioning (e.g., different cognitive or clinical domains), many types of abnormal patterns can be detected, including classical disease clusters and also disease continua that describe pathology in terms of a gradual progression rather than in terms of sharply defined clusters (see Supplementary Methods for further details).

Surprisingly few alternatives to clustering have been proposed. Proposed alternatives are of 3 main types: first, some methods extend supervised learning to classify predefined disease states while accommodating uncertainty in the class labels. This has been achieved in the following ways: embedding the algorithm in a “wrapper” that identifies mislabeled samples [(74) Figure 1A, B]; semisupervised methods that only use labels for subjects with a definite diagnosis [(75) Figure 1C]; and hybrid methods that combine supervised learning with clustering [(76, 77, 78) Figure 1D] or fusing the image registration process with FMMs such that brain images are clustered at the same time as they are registered together (79). Second, manifold learning techniques (Figure 2A) have been used to find low-dimensional representations of the data that highlight salient axes of variation. For high-dimensional data, approaches that preserve local distances are well-suited for this (80) and have been used to find latent structure underlying neurologic disorders (81) and used for dimensionality reduction before clustering (82). Third, novelty detection algorithms, such as the one-class support vector machine (83), aim to identify samples that are different from a set of training examples [(84) Figure 3B].

Normative modeling (Figure 3) is an alternative approach for parsing heterogeneity in clinical conditions (22, 85, 86) and aims to model biological variation within clinical cohorts, such that symptoms in individual patients can be recognized as extreme values within this distribution. This can be compared to the use of growth charts to map child development in terms of height and weight as a function of age, where deviations from a normal growth trajectory manifest as outliers within the normative range at each age. This is operationalized by learning some decision function that quantifies the variation across the population range, including healthy functioning and also potentially symptoms (see Supplementary Methods). Such approaches have been proposed for identifying subjects that have an abnormal maturational trajectory in brain structure (86) or in cognitive development (85), or for mapping any clinically relevant variable (22). This approach breaks the symmetry inherent in case-control and clustering approaches and provides multiple benefits. First, it does not entail making strong assumptions about the clinical group (e.g., existence or number of subgroups). This was shown by Marquand *et al.* (22), where the clinical variables did not form clearly defined clusters but normative modeling identified distinct brain mechanisms that give rise to symptoms. Second, it allows both normal functioning and deviations from normal functioning that may underlie symptoms to be mapped in individual subjects. Third, it permits diagnostic labels to be used as predictor variables, enabling inferences over the labels. Finally, it intuitively matches the clinical conception where diseases in individual patients are recognized as deviations from normal functioning. This approach can be used to estimate mappings between biology and behavior across multiple cognitive domains; therefore, it is well aligned with RDoC and ROAMER and also compliments clustering because clustering algorithms can still be applied to these mappings. On the other hand, normative modeling requires careful data processing to ensure that the outliers detected are not outliers from the normative distribution due to artifacts. It is also best suited to large normative cohorts that capture the full range of functioning in the reference population.

## Discussion

In this article, we introduced the basic concepts of data-driven stratification of psychiatric disorders and reviewed the existing literature. The overwhelming majority of studies have employed clustering or FMM, aiming to subgroup clinical populations. This has been somewhat successful (Table 1, Table 2, Table 3, Table 4, Table 5), although the results are heavily dependent on the type of data used; for most disorders, both the number and characteristics of the derived clusters vary between studies, and a consensus as to a consistent set of subgroups is yet to be reached. We highlighted the importance of validation to ensure that derived clusters map onto clinically relevant variation and outlined various alternatives to clustering.

The ongoing discussion surrounding psychiatric nosology reflects well-acknowledged difficulties in finding biological markers that predict current disease state or future outcomes with sufficient sensitivity and specificity to be clinically useful (1, 10). While this is an important motivation behind RDoC and ROAMER (11, 12, 87), this review highlights that neither the reclassification of psychiatric disorders nor the emphasis on cutting across current diagnostic classifications is a central innovative feature. A more important contribution is a shift away from symptoms and towards conceptualizing pathology as spanning multiple domains of functioning and across multiple levels of analysis. In RDoC, this is represented as a matrix with rows containing basic cognitive dimensions (“constructs”) grouped into domains of functioning (e.g., positive or negative valence systems) and columns containing units of analysis (e.g., genes, cells, or circuits) (87). Viewed in this light, clustering of algorithms provides only a partial answer to the challenges posed by RDoC and ROAMER because it does do not provide an obvious means to link constructs with units of analysis. Put simply, it is necessary to link the rows of the RDoC matrix with its columns and chart the variation in these mappings. This is necessary before the clinical validity of RDoC domains can be assessed as to whether they predict disease states more accurately than classical diagnostic categories (38).

Surprisingly few methods have been proposed that meet these objectives. Most that do exist aim to break the symmetry that both the case-control paradigm and clustering approaches entail in that all clinical groups are well-defined entities. Normative modeling (22, 85, 86) is one particularly promising approach that aims to map variation in clinically relevant variables, so that each individual subject can be placed within the population range and disease can be considered as an extreme deviation from a normal pattern of functioning. This provides a workable alternative to lumping and splitting the psychiatric phenotype and a method to chart variability across different domains of functioning and different units of analysis.

Our review also highlighted that few studies have used biological measures to derive stratifications. This may be because of difficulties that unsupervised methods have with separating nuisance variation from clinically relevant variation, particularly in high dimensions (31). This may be particularly problematic in genomic studies; some reports have used genomic data as validation of the derived clusters (60, 68), but the only study we are aware of that used genomic data to derive clusters (88) has received severe criticism for inadequately dealing with artefactual variation.^8^ One way that this problem may be addressed in the future is by developing richer clustering models that integrate clinical or domain knowledge in a way that guides the clustering algorithm toward clinically relevant variation. A simple example is the use of growth mixture models to cluster samples on the basis of within-participant change over time (39, 40). More generally, probabilistic graphic models (24) provide an elegant framework that allows existing knowledge to be incorporated to help find clinically meaningful clusters. To our knowledge, this approach has not been used in psychiatry, but it has been useful to stratify disease cohorts in other clinical domains (89). Other emerging machine learning techniques that may be fruitfully applied to stratifying psychiatric disorders include probabilistic methods that allow for multiple labels within individual patients (90), clustering methods that do not uniquely assign points to a single cluster (31), and deep learning methods (91, 92).

In summary, we reviewed the literature for stratifying psychiatric disorders and showed that the field has, to date, relied heavily on clustering and FMM. These undoubtedly provide an important contribution but only partially satisfy the objectives of RDoC and ROAMER. It is also necessary to chart variation in brain-behavior mappings to fully parse heterogeneity across domains of functioning and diagnostic categories. The hope is that using such mappings to derive future disease stratifications will enable clinical phenotypes to be dissected along the most relevant axes of variation, ultimately enabling treatments to be better targeted to individual patients.

## Figures and Tables

**Table 1:** Studies Using Clustering Methods to Stratify Schizophrenia

Study	Subjects (*N*)	Measures	Algorithm	No. of Clusters (Method)	Cluster Descriptions	External Validation
Farmer *et al*., 1983 (8)	SCZ (65)	Symptoms and case history variables	K means and hierarchical clustering	2 (maximal agreement between methods)	Good premorbid adjustment, late onset, and well organized delusions	None
Poor premorbid functioning, early onset, incoherent speech, and bizarre behavior
Castle *et al.,* 1994 (93)	SCZ (447)	Symptoms and case history variables	LCCA	3 (χ^2^ test)	Neurodevelopmental	Premorbid, phenomenologic, and treatment response variables [see (94)]
Paranoid
Schizoaffective
Dollfus *et al.,* 1996 (95)	SCZ (138)	Symptoms	Ward’s hierarchical clustering method (32)	4 (informal examination of cluster dendrogram)	Positive symptoms	Social variables
Negative symptoms
Disorganized symptoms
Mixed symptoms
Kendler *et al.,* 1998 (96)	SCZ (348)	Symptoms	LCCA	6 (not specified)	Classic schizophrenia	Historical data
Major depression
Schiophreniform disorder
Bipolar-schizomania
Hebephrenia
Murray *et al.,* 2005 (97)	SCZ (387)	“Operational criteria” diagnostic measures (medical records and interview)	LCCA	BIC (42)	Depression	None
Reality distortion
Mania
Disorganization
Dawes *et al.,* 2011 (98)	SCZ and SAD (144)	Neuropsychological measures	K means	5 (Ward method)	Visual learning and memory (–)	None
Verbal comprehension (+), processing speed (+), abstraction (–) auditory and visual learning, and memory (–)
Abstraction (–)
Verbal comprehension (+), visual learning and memory (+), abstraction (–), auditory learning and memory (–)
Verbal comprehension (+), abstraction (–), visual learning and memory (–)
Cole et al., 2012 (99)	SCZ (208)	Social and academic adjustment scales	LCGA	3 [BIC and Lo-Mendell-Rubin test (44)]	Good—stable	None
Insidious onset
Poor deteriorating
Bell *et al.,* 2013 (18)	SCZ and SAD (77 + 63 validation)	Symptoms and social cognitive measures	K means	3 (Ward method)	High negative symptoms	None
High social cognition
Low social cognition
Brodersen *et al.*, 2014 (13)	SCZ (41) and HC (42)	Dynamic causal model (100) derived from fMRI data	Gaussian mixture	3 [Bayesian model evidence (101)]	Subgroups characterized in terms of DCM model parameters	Symptoms and medication
Geisler *et al.,* 2015 (102)	SCZ (129)	Neuropsychological measures	K-means	4 (fixed a priori)	Verbal fluency (–), processing speed (–)	fMRI
Verbal episodic memory (–), fine motor control (–), signal detection
Face episodic memory (–), processing speed (–)
General intellectual function (–)
Sun et al., 2015 (52)	SCZ (113)	White matter integrity measured by diffusion tensor imaging	Hierarchical clustering	2 [Silhouette, Dunn, and connectivity indices (46, 47, 48)]	Subgroups characterized in terms of white matter abnormalities	Symptoms

External validation is defined as a data measure used to validate the derived classes that is of a different type to the data use to derive the classes. Wherever possible, we follow the authors’ own nomenclature for describing clusters, and a (+) or (–) indicates relative improvement or deficit in the specified variable.

BIC, Bayesian information criterion; DCM, dynamic causal modeling; fMRI, functional magnetic resonance imaging; LCCA, latent class cluster analysis; LCGA, latent class growth analysis; SAD, schizoaffective disorder; SCZ, schizophrenia.

**Table 2:** Studies Using Clustering Methods to Stratify Depression

Study	Subjects (*N*)	Measures	Algorithm	No. of Clusters (Method)	Cluster Descriptions	External Validation
Paykel, 1971 (7)	Patients with depression (165)	Clinical interviews, case history, and personality variables	Friedman–Rubin algorithm (103)	4 (maximize the ratio of between to within class scatter)	Psychotic	None
Anxious
Hostile
Young depressive with personality disorder
Maes *et al.,* 1992 (57)	MDD (80)	Symptoms	K means	2 (not specified)	Vital (i.e., psychomotor disorders, loss of energy, early morning awakening, and nonreactivity)	Biological (e.g., endocrine) measures
Nonvital
Kendler *et al.,* 1996 (53)	Female twin pairs (2163)	Symptoms	LCCA	7 (not specified)	Only 3 clusters described:	Body mass index, personality, and concordance of cluster membership among twin pairs
Mild typical depression Atypical depression Severe typical depression
Sullivan *et al.,* 1998 (54)	National comorbidity survey respondents (2836)	Symptoms	LCCA	6 (χ^2^ statistic)	Severe typical	Demographic and personality variables
Mild typical
Severe atypical
Mild atypical
Intermediate
Minimal symptoms
Hybels *et al.,* 2009 (58)	MDD (368)	Symptoms	LCCA	4 [*L*^2^ statistic (34), BIC]	DSM-IV depression: Moderate sadness, lassitude and inability to feel	Demographic, social, and clinical variables
Higher severity for all items, especially apparent sadness
Milder profile
Highest severity and most functional limitations
Lamers *et al.,* 2010 (55)	MDD (818)	Symptoms plus demographic, psychosocial, and physical health variables	LCCA	3 [BIC and AIC (43)]	Severe melancholic (decreased appetite, weight loss)	Stability over time, sociodemographic, clinical, and biological (e.g., metabolic) variables (104, 105)
Severe atypical (overeating and weight gain)
Moderate severity
Lamers *et al.,* 2012 (56)	National comorbidity survey—replication respondents. Adolescents (912) and adults (805)	Symptoms	LCCA	Adolescents: 3, adults: 4 (BIC)	Adolescents:	None
Moderate typical Severe typical Severe atypical
Adults:
Moderate Moderate typical Severe typical Severe atypical
Rhebergen *et al.,* 2012 (39)	MDD (804)	Longitudinal symptom scores	LCGA	5 (BIC and Lo-Mendell-Rubin test)	Remission	Demographic and diagnostic variables, fMRI [see (73)]
Decline (moderate severity)
Decline (severe)
Chronic (moderate severity)
Decline (severe)
Van Loo *et al.,* 2014 (59)	MDD (8,261)	Retrospective symptom reports and demographic data that predict disease course	K-means	3 (Inspection of dichotomization scores and area under the receiver operating characteristic curve [see (59)])	High risk	None
Intermediate risk
Low risk
Milaneschi *et al.,* 2015 (60)	MDD (1477)	Symptoms	LCCA	3 (BIC, AIC, and likelihood ratio test)	Severe melancholic [see Lamers *et al.,* (55)]	Polygenic risk scores
Severe atypical
Moderate

External validation is defined as a data measure used to validate the derived classes that is of a different type to the data use to derive the classes. Wherever possible, we follow the authors’ own nomenclature for describing clusters.

AIC, Akaike information criterion; BIC, Bayesian information criterion; fMRI, functional magnetic resonance imaging; LCCA, latent class cluster analysis; LCGA, latent class growth analysis; MDD, major depressive disorder.

**Table 3:** Studies Using Clustering Methods to Stratify Attention-Deficit/Hyperactivity Disorder

Study	Subjects (*N*)	Measures	Algorithm	No. of Clusters (Method)	Cluster Descriptions	External Validation
Fair *et al.,* 2012 (15)	ADHD (285) and TDC (213)	Neuropsychologic scores	CD (33)	6 for ADHD (determined implicitly by the algorithm)	Response time variability (+)	None
Working memory (–), memory span (–), inhibition (–), and output speed (–)
Working memory (–), memory span (–), inhibition (–), and output speed (–), minor differences in remaining measures
Temporal processing (–)
Arousal (–)
Arousal (–), minor differences in remaining measures
Karalunas *et al.,* 2014 (14)	ADHD (247) and TDC (190)	Personality measures (e.g., temperament)	CD	3 (determined implicitly by the algorithm)	Mild	Physiological (e.g., cardiac) measures, resting state fMRI and 1-year clinical outcomes
Surgent (positive apporach motivation)
Irritable (negative emotionality, anger, and poor soothability)
Gates *et al.,* 2014 (16)	ADHD (32) and TDC (58)	fMRI (functional connectivity)	CD	5 (determined implicitly by the algorithm)	Subgroups characterized in terms of functional connectivity profiles	None
Costa Dias *et al.,* 2015 (17)	ADHD (42) and TDC (63)	fMRI (reward related functional connectivity)	CD	3 (determined implicitly by the algorithm)	Subgroups characterized in terms of functional connectivity profiles	Clinical variables and reward sensitivity
Van Hulst *et al.,* 2015 (67)	ADHD (96) and TDC (121)	Neuropsychological scores	LCCA	5 (BIC)	Quick and accurate	Parent ratings of behavioral problems
Poor cognitive control
Slow and variable timing
Remaining 2 groups were too small to characterize
Mostert *et al.,* 2015 (106)	ADHD (133) and TDC (132)	Neuropsychological scores	CD	3 (determined implicitly by the algorithm)	Attention (–), inhibition (–)	Clinical symptoms and case history
Reward sensitivity (+)
Working memory (–) and verbal fluency (–)

External validation is defined as a data measure used to validate the derived classes that is of a different type to the data use to derive the classes. Wherever possible, we follow the authors’ own nomenclature for describing clusters, and a (+) or (–) indicates relative improvement or deficit in the specified variable.

ADHD, attention-deficit/hyperactivity disorder; BIC, Bayesian information criterion; CD, community detection; fMRI, functional magnetic resonance imaging; LCCA, latent class cluster analysis; TDC, typically developing control.

**Table 4:** Studies Using Clustering Methods to Stratify Autism

Study	Subjects (*N*)	Measures	Algorithm	No. of Clusters (Method)	Cluster Descriptions	External Validation
Munson *et al.,* 2008 (107)	ASD (245)	IQ scores	LCCA and taxonometric analysis	4 (BIC, entropy, and Lo-Mendell-Rubin test)	Low IQ	Symptom scores
Low verbal IQ/medium nonverbal
Medium IQ
High IQ
Sacco *et al.,* 2012 (21)	ASD (245)	Demographic, clinical, case history, and physiologic (e.g., head circumference) variables	K means	4 (Ward’s method)	Immune + circadian and sensory	None
Circadian and sensory
Stereotypic behaviors
Mixed
Fountain *et al.,* 2012 (40)	ASD (6795)	Symptoms	LCGA	6 (BIC)	High functioning	Demographic variables and autism risk factors
Bloomers (substantial improvement)
Medium-high functioning
Medium functioning
Low-medium functioning
Low functioning
Georgiades *et al.,* 2013 (108)	ASD (391)	Symptom scores	FMM	3 (AIC and BIC)	Social communication (–), repetitive behaviors (+)	Demographic and cognitive meaures
Social communication (+), repetitive behaviors (–)
Social communication (–), repetitive behaviors (–)
Doshi-Velez *et al.,* 2014 (109)	ASD (4927)	Electronic medical records	Ward’s method	4 (Ward’s method)	Seizures	None
Multisystem disorders
Auditory disorders and infections
Psychiatric disorders
Not otherwise specified
Veatch *et al.,* 2014 (68)	ASD (1261 + 2563 for replication)	Symptoms, demographic, and somatic variables	Ward’s method	2 [Adjusted Arabie Rand index (110) and validation with additional clustering algorithms]	Severe	Genomic data
Less severe

External validation is defined as a data measure used to validate the derived classes that is of a different type to the data use to derive the classes. Wherever possible, we follow the authors’ own nomenclature for describing clusters, and a (+) or (–) indicates relative improvement or deficit in the specified variable.

ASD, autism spectrum disorder; BIC, Bayesian information criterion; FMM, factor mixture modeling; LCCA, latent class cluster analysis; LCGA, latent class growth analysis.

**Table 5:** Studies Employing Clustering Methods to Stratify Patients in a Cross-Diagnostic Setting

Study	Subjects (*N*)	Measures	Algorithm	No. of Clusters (Method)	Cluster Descriptions	External Validation
Olinio *et al.,* 2010 (113)	Adolescents (1653), including MDD (603), ANX (253), SUD (453)	Diagnosis (longitudinal)	LCGA	6 (BIC)	Persistent depression	Demographic and case history variables
Persistent anxiety
Late onset anxiety, increasing depression
Increasing depression
Initially high, decreasing anxiety
Absence of psychopathology
Lewdanowski *et al.,* 2014 (111)	SCZ (41), SAD (53), BPDp (73)	Clinical and cognitive measures	K means	4 (Ward’s method)	Neuropsychologically normal	Diagnosis, demographic variables, and community functioning
Globally and significantly impaired
Mixed cognitive profiles (×2)
Kleinman *et al.,* 2015 (112)	ADHD (23), BPD (10), BPDa (33), and HCs (18)	Continuous performance test measures	K means	2 [Silhouette index (46)]	Sustained attention (–) , inhibitory control (–), impulsiveness (+), and vigilance (–)	Diagnosis
The converse of above

External validation is defined as a data measure used to validate the derived classes that is of a different type to the data use to derive the classes. Wherever possible, we follow the authors’ own nomenclature for describing clusters and a (+) or (–) indicates relative improvement or deficit in the specified variable.

ADHD, attention-deficit/hyperactivity disorder; ANX, anxiety disorders; BPD(p/a), bipolar disorder (with psychosis/ADHD); BIC, Bayesian information criterion; DEP, depressive disorders (major depression and dysthymia); HC, healthy control; LCGA, latent class growth analysis; MDD, major depressive disorder; SAD, schizoaffective disorder; SCZ, schizophrenia; SUD, substance use disorder.

## Footnotes

1 We identified studies by performing a PubMed search for each disorder separately using the following search string: [(clustering OR subtypes OR subgroups OR stratification) and (disorder name OR disorder acronyms)]. We then selected a representative overview of studies for each disorder (this was exhaustive for ADHD, autism, and cross-diagnostic studies). For example, in the case of multiple studies using the same cohort, we only included the first or most important in this review. We also gave priority to studies that have not been reviewed previously (19, 51, 52).

2 Many of the FMM approaches discussed here originate in the psychometric literature, which uses different nomenclature to mainstream statistics. Unfortunately, this nomenclature also varies between authors. We use consistent terminology throughout and synthesize with mainstream statistical literature wherever possible.

3 Referred to as “latent profile analysis” in the psychometric literature.

4 Also referred to as “group-based trajectory modeling.”

5 The overall objectives of clustering approaches and FMMs are similar; for the remainder of this article, we refer to both as “clustering” for brevity.

6 Technically, clustering belongs to the “NP-hard” class of problems.

7 In contrast, there is a clear measure by which success of supervised methods can be assessed: the expected loss, measured by some loss function, over the joint distribution of labels and covariates. This can be estimated in various ways (e.g., cross-validation).

8 For example, see the discussion at: http://www.ncbi.nlm.nih.gov/pubmed/25219520.
