# Supplementary material for: Beyond Lumping and Splitting: A Review of Computational Approaches for Stratifying Psychiatric Disorders
Source: Biol Psychiatry Cogn Neurosci Neuroimaging. 2016 Sep;1(5):433–47. doi: 10.1016/j.bpsc.2016.04.002 (PMC5013873; doi:10.1016/j.bpsc.2016.04.002)
Supplement: Supplementary file 1 — Supplementary material [file mmc1.pdf]

Beyond Lumping and Splitting: A Review of Computational Approaches for Stratifying Psychiatric Disorders

Supplemental Information

Supplementary Methods

Clustering

Given a set of D-dimensional data points, $\{\mathbf{x}_i\}_{i=1}^{N}$ (e.g., clinical or neuroimaging measures), clustering algorithms aim to partition the data into a specified number (K) of clusters such that the samples in each cluster are more similar to one another than to those in the other clusters. One of the simplest and most widely used approaches for clustering is the 'K-means' algorithm, which is an iterative approach involving two steps: (i) for each data point, find the closest cluster center according to the squared Euclidean distance, $d(\mathbf{x}_i, \mathbf{x}_j) = \|\mathbf{x}_i - \mathbf{x}_j\|^2$, then (ii) replace each center with the coordinate wise mean of all points assigned to it. These steps are iterated until the cluster assignments do not change.

Finite Mixture Modeling

Conventionally, FMMs are specified using a linear superposition of component distributions:

$$p(\mathbf{x}) = \sum_{k=1}^{K} \pi_k p_k(\mathbf{x}|\boldsymbol{\theta}_k)$$

Where K denotes the number of components (model order), $p_k(\mathbf{x}|\boldsymbol{\theta}_k)$ is the distribution for each component (parameterized by $\boldsymbol{\theta}_k$) and π_k are mixing coefficients specifying the proportion each component contributes to the mixture, such that $0 < \pi_k < 1$ and $\sum_k \pi_k = 1$. For a GMM, each component is Gaussian with $\boldsymbol{\theta}_k$ specifying a mean and covariance. Gaussian mixture models are related to K-means (1) but provide soft cluster assignments, determined by the covariance of the component distributions. This can be beneficial in the case of noisy or overlapping clusters (see Figure S1, below), but necessitates estimating parameters to characterize each component. There

are a range of approaches for fitting FMMs but maximum likelihood estimation using the expectation-maximization algorithm is most common (1, 2).

Latent class cluster analysis is a widely used FMM approach that accommodates many different data types. In its simplest form, LCCA assumes independence between variables, i.e.:

$$p(\mathbf{x}) = \sum_{k=1}^{K} \pi_k \prod_{d=1}^{D} p_k(x_d|\boldsymbol{\theta}_{dk})$$

Here, the components, $p_k(x_d|\boldsymbol{\theta}_{dk})$, are Gaussian, binomial, multinomial, ordered multinomial or Poisson distributions depending on the data type (continuous, categorical, ordinal or count). There are also many generalizations: for example, the independence assumption can be relaxed (e.g., to model clinical variables that may be correlated) and covariates can be introduced to help predict class membership (3-5). Many classical FMM techniques can be seen as generalizations of LCCA (see main text).

Didactic Example of Clustering Methods

We show a toy dataset (Figure S1A) to illustrate the behavior of K-means (Figure S1B) and a GMM (Figure S1C). The data were generated from four Gaussian distributions chosen deliberately so that two of the classes are relatively distinct but the remaining two classes overlap. This illustrates: (i) the partitioning of the input space induced by K-means (a 'Voronoi tessellation') and (ii) the soft assignment performed by the GMM along with its underlying probability contours. Note that neither approach faithfully represents the true model: K-means performs a hard partitioning of the data and cannot accommodate class overlap, whereas the GMM does not learn the correct distribution for the overlapping classes. Moreover, two common measures to select model order (6, 7) favor a simpler model having only three clusters relative to the true model (Figure S1D). Thus, more data are required to identify the overlapping classes.

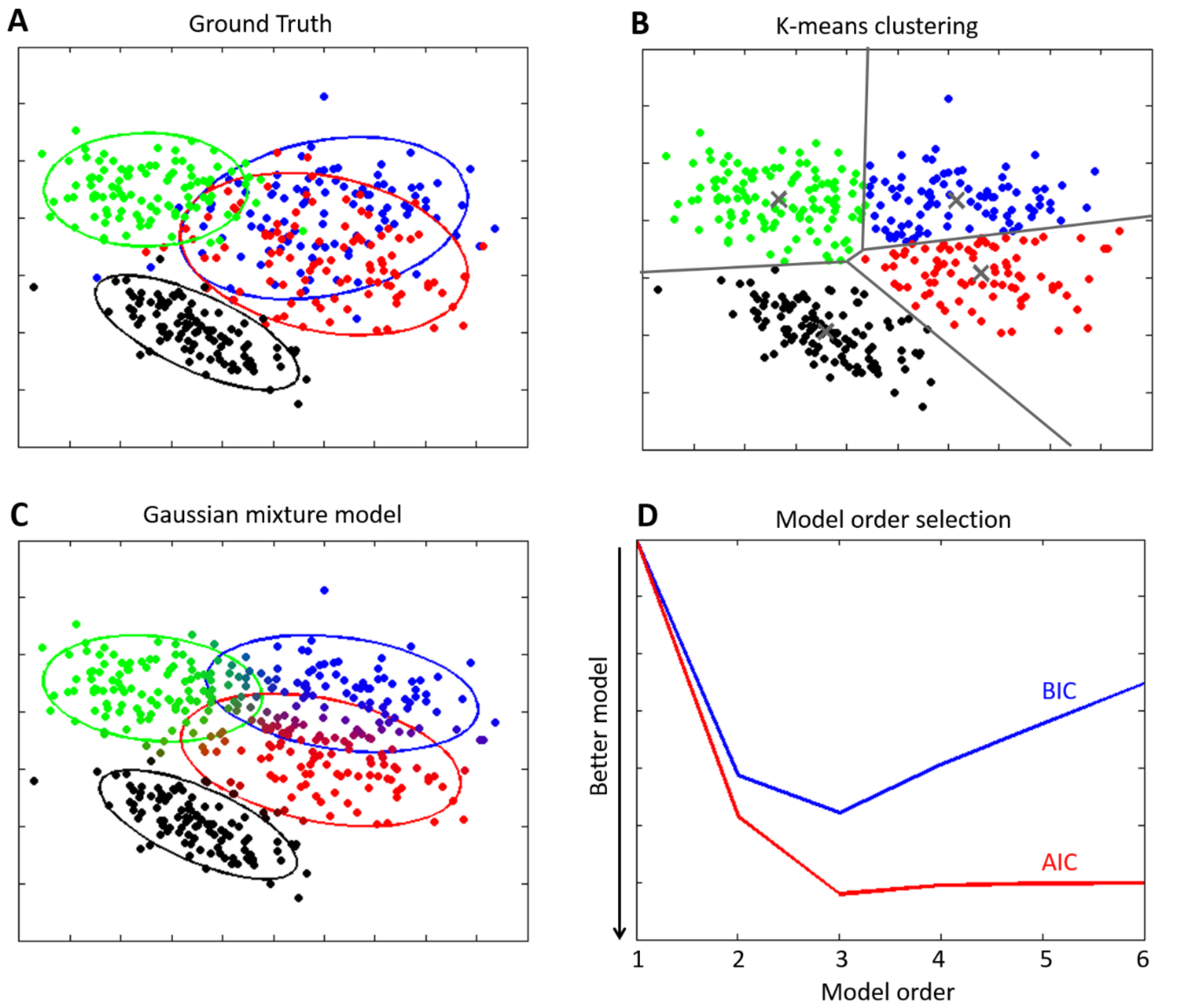

Figure S1. A didactic example illustrating the operation of k-means clustering and a Gaussian mixture model. **(A)** A two-dimensional dataset was generated from four Gaussian distributions, each containing 100 data points. These are shown in different colors with the elliptical contours denoting 2 standard deviations. The mean and covariance of these distributions were chosen such that two clusters are relatively easily to discriminate (black and green) while two clusters are more difficult to discriminate because they overlap substantially (red and blue). **(B)** The results of the K-means clustering algorithm estimated using good practice (e.g., 10 random restarts). Cluster centers are denoted by gray crosses and the borders between each class by lines. **(C)** The results of applying a Gaussian mixture model with 4 components to the same data. Each point is colored according to a mixture of the posterior probabilities of belonging to each class (see 1). **(D)** The results of applying two common approaches to select the model order, the Akaike information criterion (AIC; 7) and the Bayesian information criterion (BIC; 6). Both measures favor a simpler three cluster model over the true data generating model (i.e., with four clusters). See text for further details.

Clustering in High Dimensions

Clustering is well-known to be a notoriously difficult problem in high dimensional spaces (8, 9) for many reasons: conventional distance measures are typically not meaningful in high dimensions; the presence of many noisy features or correlations between features strongly influences the derived

clusters; different features may be relevant for identifying different clusters, or different clusters may lie on different subspaces. Moreover, most conventional feature selection or dimensionality reduction techniques (e.g., principal components analysis) act globally in that they generate a single subspace for all the data, so they have limited value in alleviating this problem.

High-dimensional clustering is a large field in machine learning, and the fact that there is no universal measure of clustering success has led to a heavy proliferation of different approaches (see (10) and the main text). Therefore, only a very brief introduction will be given here (see (8) for a more extensive treatment). Briefly however, the clustering algorithm must solve two separate problems in high dimensions. First, it must find the relevant subspaces that best explain the data and then must identify what the final clusters in these subspaces are (akin to the main problem that must be solved in low dimensions). These are both extremely difficult problems and all existing algorithms invoke heuristics for both. For example, methods to find relevant subspaces for the data might assume that they have a simple structure (e.g., being axis aligned), they might project the data onto multiple subspaces or might not try to assign each data point uniquely to a single cluster at all (referred to as 'hybrid' algorithms (8)). A particularly popular approach in biostatistics is 'biclustering' (11) which aims to cluster both the features and samples simultaneously. To date none of these approaches have been employed to stratify psychiatric disorders.

Alternatives to Clustering: Normative Modeling

Normative modeling differs from clustering in that it aims to learn mappings between clinically relevant variables and quantitative biological variables across the full range of variation. This allows disease effects to be detected in individual subjects either: (i) as outliers from the normative model (i.e., having patterns of variation that are distinct from the normative cohort) or (ii) by being towards the extreme end of a normal axis of variation but still well-explained by the normative model (see below). The approach is highly generic and is suitable for high dimensional data (e.g., whole-brain voxel-wise data (12, 13)) and also to multi-modal data. The first step is to fit a normative distribution

that links clinically relevant variables with quantitative biological variables using a large cohort (e.g. consisting of healthy subjects). This has been achieved in different ways and using different multivariate regression techniques: Erus and colleagues (12) used support vector regression (14) to predict chronological age from a set of structural neuroimaging measurements in a 'decoding' model (15), yielding a 'brain developmental index'. They then identified outliers as subjects who were beyond the 90% confidence interval band from a linear regression between this measure and true chronological age. Marquand and colleagues (13) adopted an alternative 'encoding' model, where they used a probabilistic regression method (Gaussian process regression; 16) to predict regional brain activity from clinically relevant covariates. An advantage of Gaussian process regression is that it automatically provides measures of predictive confidence that quantify where each subject lies in the normal range for each of the biological response variables, which means that a second linear regression step is not necessary. Under this encoding approach, a separate normative model is estimated for each brain region which together quantify an individual participant's response pattern, given their clinical or behavioral covariates and provide a natural approach for mapping brain regions that differ from the normative cohort at the individual subject level (17). In the case of a multivariate response (e.g., having thousands of brain locations) this can be considered as a multivariate measure of deviation. For subject-specific decision-making it is necessary to summarize the degree of abnormality by estimating the total magnitude of deviation for each subject with respect to the normative model. This can be achieved using extreme value statistics, which model the behavior of random variables in the tail of their distribution (18, 19). Briefly, an extreme value distribution can be fit to the multivariate response patterns across subjects to derive a subject-level abnormality score.

In a second step, the normative model can be applied to new subjects; which can be: (i) a withheld part of the original cohort (e.g., under cross-validation); (ii) a second cohort containing healthy subjects, or (iii) a subset of subjects that express symptoms. This allows disease effects in the target population to be assessed with respect to the normative distribution. These effects may

manifest as outliers within the distribution, but not necessarily so. It is possible that the normative distribution covers a sufficiently wide amount of variation that it also explains the range of functioning that drives symptom expression. In such cases, the normative model can be interrogated to determine which ranges of the clinical predictor variables give the most important contribution to symptom expression. Finally, the fit of each individual subject to the normative model can be used as input to a clustering algorithm to define subtypes. This provides two potential advantages over clustering the data directly: (i) the mapping between biology and behavior may be more informative than either individually for separating subtypes and (ii) the subtypes are defined with respect to a healthy pattern of functioning, and are therefore potentially more interpretable. See (13) for further details on normative modeling.

Supplementary References

1. Bishop C (2006): *Pattern Recognition and Machine Learning*. Springer.
2. Dempster AP, Laird NM, Rubin DB (1977): Maximum Likelihood From Incomplete Data Via Em Algorithm. *Journal of the Royal Statistical Society Series B-Methodological*. 39:1-38.
3. Hagenaars JA, McCutcheon AL (2002): *Applied Latent Class Cluster Analysis*. Cambridge: Cambridge University Press.
4. Muthen B (2002): Beyond SEM: General Latent Variable Modeling *Behaviormetrika*. 29:81-117.
5. Lazarsfeld PF, Henry NW (1968): *Latent Structure Analysis*. Boston: Houghton Mifflin.
6. Schwarz G (1978): Estimating Dimension Of A Model. *Annals of Statistics*. 6:461-464.
7. Akaike H (1974): A new look at the statistical model identification. *IEEE Transactions on Automatic Control*. 19:716-723.
8. Kriegel H-P, Kroeger P, Zimek A (2009): Clustering High-Dimensional Data: A Survey on Subspace Clustering, Pattern-Based Clustering, and Correlation Clustering. *Acm Transactions on Knowledge Discovery from Data*. 3.
9. Bouveyron C, Brunet-Saumard C (2014): Model-based clustering of high-dimensional data: A review. *Computational Statistics & Data Analysis*. 71:52-78.
10. Hastie T, Tibshirani R, Friedman J (2009): *The Elements of Statistical Learning*. 2nd ed. New York: Springer.
11. Madeira SC, Oliveira AL (2004): Biclustering Algorithms for Biological Data Analysis: A Survey. *IEEE Transactions on Computational Biology and Bioinformatics*. 1:24-45.
12. Erus G, Battapady H, Satterthwaite TD, Hakonarson H, Gur RE, Davatzikos C, et al. (2015): Imaging Patterns of Brain Development and their Relationship to Cognition. *Cerebral Cortex*. 25:1676-1684.
13. Marquand AF, Rezek I, Buitelaar J, Beckmann CF (2016): Understanding heterogeneity in clinical cohorts using normative models: beyond case control studies. *Biological Psychiatry*. in press.
14. Drucker H, Burges CJC, Kaufman L, Smola A, Vapnik V (1997): Support vector regression machines. *Advances in Neural Information Processing Systems 9*. 9:155-161.
15. Naselaris T, Kay KN, Nishimoto S, Gallant JL (2011): Encoding and decoding in fMRI. *NeuroImage*. 56:400-410.
16. Rasmussen CE, Williams C (2006): *Gaussian Processes for Machine Learning*. MIT Press.
17. Ziegler G, Ridgway GR, Dahnke R, Gaser C, Alzheimer's Dis N (2014): Individualized Gaussian process-based prediction and detection of local and global gray matter abnormalities in elderly subjects. *Neuroimage*. 97:333-348.
18. Beirlant J, Goegebeur Y, Teugels J, Segers J (2004): *Statistics of Extremes: Theory and Applications*. Sussex, England: John Wiley and Sons.
19. Coles S (2001): *An Introduction to Statistical Modeling of Extreme Values*. Springer.
